# Successful rescue of a lethal Griscelli syndrome type 2 presenting with neurological involvement and hemophagocytic lymphohistiocytosis: a case report

**DOI:** 10.1186/s12887-021-02720-1

**Published:** 2021-05-31

**Authors:** Qing Zhang, Yun-Ze Zhao, Hong-Hao Ma, Dong Wang, Nan Zhang, Zhi-Gang Li, Rui Zhang

**Affiliations:** 1 Hematologic Disease Laboratory; Hematology Center; Beijing Key Laboratory of Pediatric Hematology Oncology; National Key Discipline of Pediatrics (Capital Medical University); Key Laboratory of Major Diseases in Children, Ministry of Education; Beijing Pediatric Research Institute;, Beijing Children’s Hospital, Capital Medical University, National Center for Children’s Health, 100045 Beijing, China; 2 Hematology Center; Beijing Key Laboratory of Pediatric Hematology Oncology; National Key Discipline of Pediatrics (Capital Medical University); Key Laboratory of Major Diseases in Children, Ministry of Education; Beijing Children’s Hospital, Capital Medical University, National Center for Children’s Health, 100045 Beijing, China; 3 Department of Pathology, Beijing Children’s Hospital, Capital Medical University, National Center for Children’s Health, 100045 Beijing, China

**Keywords:** Griscelli syndrome type 2, *RAB27A*, Hemophagocytic lymphohistiocytosis, Central nervous system involvement

## Abstract

**Background:**

Griscelli syndrome type 2 (GS2) is a rare autosomal recessive disease caused by mutations in *RAB27A* gene. It is primarily characterized by a combination of partial albinism, hemophagocytic lymphohistiocytosis (HLH) or other immunodeficiency. However, neurological involvement at onset in GS2 and treatment has rarely been described.

**Case presentation:**

We describe a 3-year-old boy with GS2 in an Asian Chinese family. He presented with progressive neurological abnormalities following unremitting fever at onset. He developed HLH during the clinical course. A novel homozygous mutation (c.1 A > G) in *RAB27A* gene was subsequently identified. He was then treated by HLH-1994 protocol combined with ruxolitinib and experienced a dramatic remission. He subsequently underwent a successful haploidentical hematopoietic stem cell transplantation and stayed at a good condition.

**Conclusions:**

We reported an atypical form of GS2 manifesting as severe central nervous system involvement at onset and subsequent HLH, which was successfully rescued in time. This case also highlights the need for early consideration of immunologic and genetic evaluation for HLH in unexplained neuroinflammation in the diagnostic work up.

## Background

Hemophagocytic lymphohistiocytosis (HLH) is a systemic hyperinflammatory syndrome characterized by unremitting fever, cytopenias, hepatosplenomegaly, elevation of typical biomarkers, and sometimes central nervous system (CNS) involvement. Primary HLH is associated with genetic defects, including familial HLH genes, *PRF1* (OMIM:603,553), *UNC13D* (OMIM:608,898), *STXBP2* (OMIM:613,101), and *STX11* (OMIM:603,552) and X-linked lymphoproliferative disease genes, *SH2D1A* (OMIM:308,240) and *XIAP* (OMIM:300,635). Genes involved in granule/pigment transport were also involved, including *RAB27A* (OMIM:607,624), *LYST* (OMIM:214,550), and *AP3B1*(OMIM:608,233), in addition to other genes such as *NLRC4* (OMIM:616,115), *CDC42* (OMIM:116,952) [1, 2]. The proteins encoded by these genes have all been involved in lymphocyte cytolytic activity. Primary HLH is often rapidly fatal, and the only curative therapy is hematopoietic stem cell transplantation (HSCT).

 Griscelli syndrome (GS) is an autosomal recessive disease with 3 distinct subtypes. Mutations in *MYO5A* (OMIM:214,450), *RAB27A* (OMIM:607,624) and *MLPH* (OMIM:609,227) genes are responsible for the differing manifestations of type 1, 2 and 3 respectively [3–5]. Partial albinism affecting the hair and skin is common to all the 3 types with additional features according to genetic involvement. Among the GS subtypes, Gricelli syndrome type 2 (GS2), caused by the mutation in *RAB27A* gene, is primarily associated with immunological dysfunction and the development of HLH [5–7]. *RAB27A*, encodes RAB27A, which is a member of the small GTPase family of proteins involved in vesicular fusion and trafficking. RAB27A is essential for the distribution of pigment-containing melansomes in melanocytes and release of cytolytic granules from T cells and natural killer (NK) cells [5, 8]. Therefore, *RAB27A* controls secretion in two different cell types by interacting with different effector proteins.

Here, we report successful rescue of a male patient from China with a novel homozygous mutation in *RAB27A* associated with an atypical form of GS2 manifesting as HLH and marked neurological abnormities at onset. The purpose of this report is to improve recognition of this uncommon, life-threatening condition and to avoid possible pitfalls.

## Case presentation

A 3-year-old boy, born out of nonconsanguineous marriage, presented with high grade intermittent fever for about 1.5 months. He was repeatedly treated with broad-spectrum antibiotics, but continued to have progressive headache without improvement for 8 days.

**Fig. 1:**
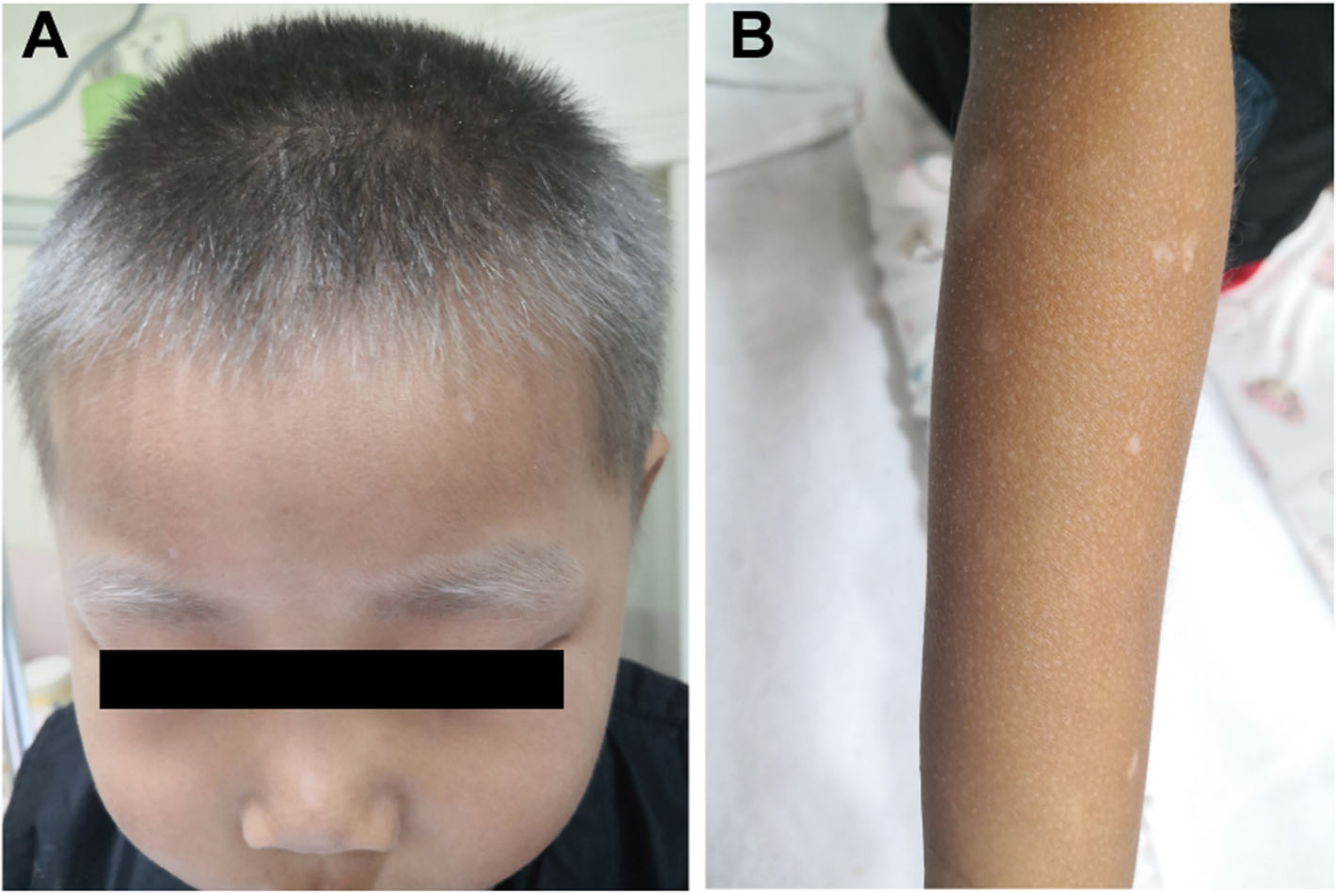
**A** silvery-grey hair of the scalp, eyebrows, and eyelashes in the patient; **B** multiple, well-defined, dot-like hypopigmented patches in his limbs and generalized dark, dry and coarse skin

The patient was then transferred to our hospital with decreased mental status, appetite and physical strength. The patient was noted to have silvery-grey hair of the scalp, eyebrows, and eyelashes, which had been present since birth (Fig. 1A). All his limbs showed multiple, well-defined, dot-like hypopigmented patches with a generalized dark, dry and coarse skin (Fig. 1B). He had no developmental delays, and received vaccinations on time without abnormalities. He has an older half-sister and his mother has no history of abortion. All his parents and half-sister are in good health, and have no family history of genetic diseases.

**Fig. 2:**
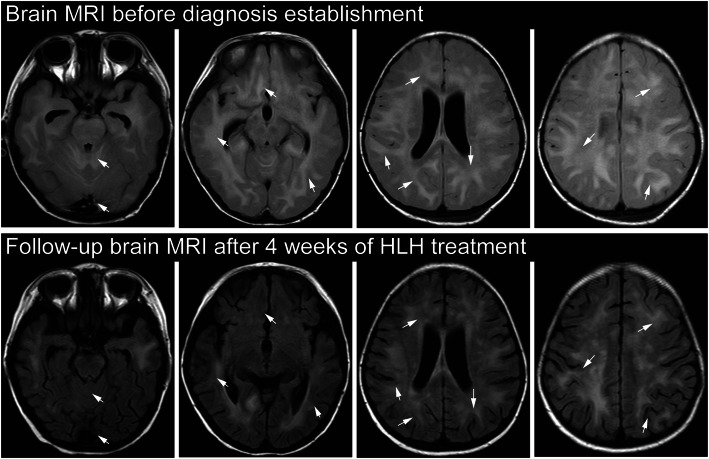
Brain MRI presentation of neurological abnormalities in fluid-attenuated inversion recovery (FLAIR) images. Brain MRI before diagnosis establishment (top row) showed extensive hyperintense lesions involving the bilateral cerebrum, cerebellum, brainstem, basal ganglia, thalamus and corpus callosum, with nodular enhancement (arrow). Follow-up brain MRI after 4 weeks of HLH treatment (bottom row) showed that most of the lesions were attenuated (arrow), and especially the original hyperintense lesions in cerebellar hemisphere and genu of corpus callosum disappeared

At admission, blood routine test was generally normal except for decreased hemoglobin (82 g/L). Meanwhile, his serum iron (4.5 µmol/L) was lower than normal, and he had been diagnosed as iron deficiency anemia prior to his admission. Erythrocyte sedimentation rate was slightly elevated at 20 mm/h. The level of C-reative protein (CRP), procalcitonin, coagulation function, ferritin, serum electrolytes, uric acid, kidney and liver function tests were within normal limits. Tests to rule out common infections like hepatitis A/B/C, HIV-1, EBV, CMV, tuberculosis, salmonella typhosa and mycoplasma pneumoniae, didn’t reveal any abnormalities. Immunoglobulin IgE was at high level of 1040 IU/ml. Autoimmune work-up, such as anti-dsDNA and antinuclear antibody (ANA), were negative. Cervical ultrasound revealed multiple lymph nodes enlargement on both sides, the largest of which was 2.2 × 0.9 cm. Besides, ultrasound of the abdomen showed hepatosplenomegaly (liver, 2 cm below costal margin; spleen, 4 cm below costal margin). The CT scan of chest showed emphysema in the left lobe, but no obvious abnormalities in abdomen and head. Bone marrow aspiration smear and biopsy examination were normal without hemophagocytosis. The cerebrospinal fluid (CSF) examination revealed increased CSF pressure (230mmH_2_O), white blood cell (WBC, 30$\times$10^6^/L, 44 % monocytes) and protein (743 mg/L) but the infection and tumor tests were negative. His first brain magnetic resonance imaging (MRI) one month before hospitalization was normal, but MRI ten days after admission showed tonsillar hernia and extensive areas of increased signal involving the bilateral cerebrum, cerebellum, brainstem, basal ganglia, thalamus and corpus callosum, with dot-like and nodular enhancement (Fig. 2). At this point, the laboratory findings were not consistent with infection, malignancy or autoimmune disease. Unifying diagnosis could not be made.

Based on the CSF findings and neurological manifestations, the patient was treated with mannitol (5ml/kg, q8h) and glycerin fructose (5ml/kg, q12h) to decrease intracranial pressure. However, the intracranial pressure remained above 330 mmH_2_O with WBC, protein and glucose elevation. His clinical status progressively deteriorated after 10 days of therapy, including daily fevers, persistent headache, worsening drowsiness, growing irritability, nuchal rigidity and insensitive pupilary light reflex. Repeat MRI demonstrated cerebral oedema. Therefore, he was administered pulse methylprednisolone (10 mg/kg/day) for 3 days to control inflammation. A possibility of central nervous system (CNS) HLH was considered. Subsequently, he presented cytopenias affecting 2 cell lineages (hemoglobin, 76 g/L; platelets, 89$\times$10^6^/L), hypertriglyceridemia (3.51 mmol/L) and hypofibrinogenemia (1.24 g/L). Soluble CD25 (sCD25) was elevated (131,840 pg/ml vs. 6400 pg/ml in control). Immunological work-up showed low NK cell activity (12.81 % vs. 15.44 % in control) and defective CD107a degranulation (1.01 % vs. 10 % in control). Serum cytokines demonstrated very high levels of IFN-γ(213.6 pg/ml) and IL-10 (50.42 pg/ml). The collective results were consistent with a diagnosis of CNS HLH. All of his laboratory and clinical findings before and at the time of diagnosis were summarized in Table 1.

**Table 1:** Patient’s laboratory and clinical findings during his clinical course

	Day 0 (at admission)	Day 13 (at HLH diagnosis)	At the 6th week after HLH treatment	Reference Range
**Parameters for HLH diagnosis**				
High fever	+	+	-	-
Splenomegaly (under ribs, cm)	4	4.5	-	-
Neutrophil count (×10^9^/L)	3.21	1.75	6.08	0.72–4.6
Platelet count (×10^9^/L)	139	89	116	100–400 × 10^9^
Hemoglobin (g/L)	82	76	284	110–190
Fibrinogen (g/L)	2.2	1.24	2.44	2–4
Triglyceride (mmol/L)	1.52	3.51	1.31	0–2
Serum ferritin (µg/L)	175.7	269.6	163	28–397
Soluble CD25(pg/mL)	ND	131,840	4117	0–6400
Hemophagocytosis in BM	-	ND	ND	-
NK cell activity (%)	ND	12.81	14.32	>15.44
**Other indicators**				
Neurological manifestations	+	+, deterioration	-	-
CSF pressure (mmH_2_O)	230	>330	ND	40–100
CSF cell count (×10^6^/L)	30	45	ND	0–15 × 10^6^
CSF Protein (mg/L)	743	1216	ND	20–350
Head CT scan	Low density of brain parenchyma	Extensive lesions, cerebral edema, tonsil hernia	Greatly improved, cerebral edema (-) tonsil hernia (-)	-
IFN-γ(pg/ml)	ND	213.6	9.83	1.60–17.30
NK cell CD107a (%)	ND	1.01	2.33	>10
**Infection, malignancy or autoimmune disease**	-	-	ND	-

+: positive; -: negative

*ND*not done

**Fig. 3:**
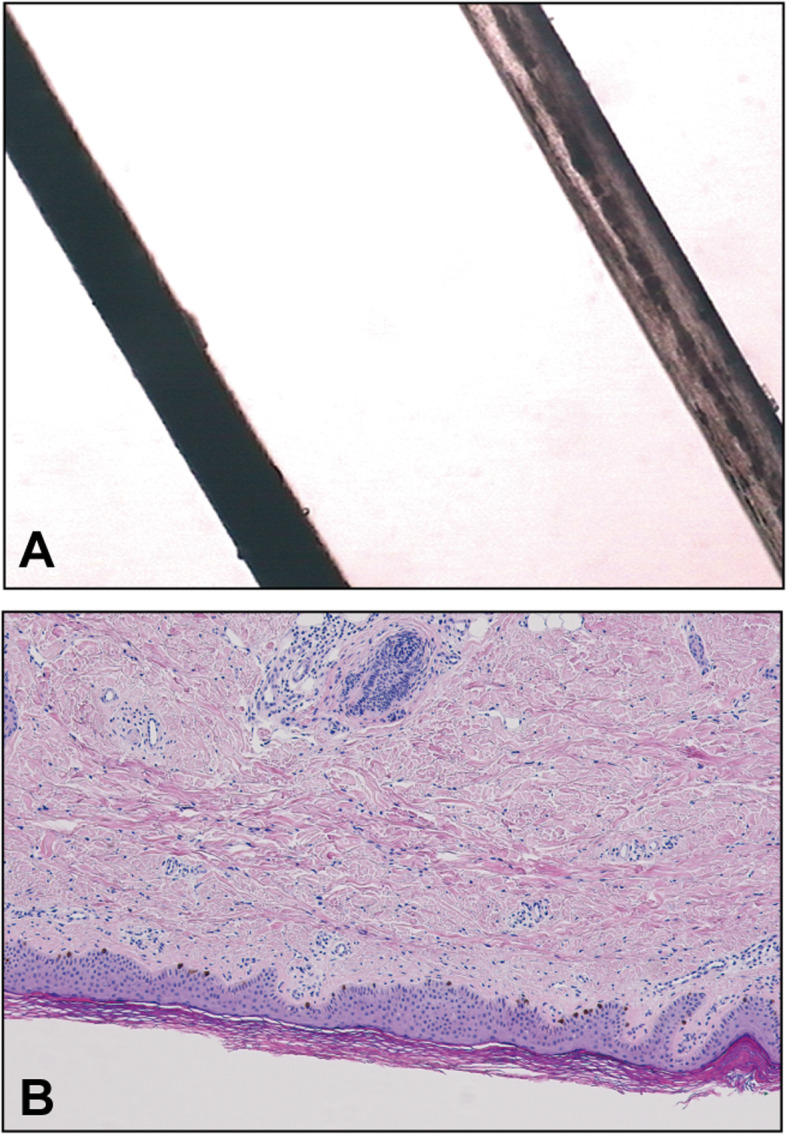
**A** compared to the healthy control (left), irregular melanosome clumping is seen in hair shafts from the patient (right) by light microscopic examination. **B** The skin biopsy was generally normal. Hyperkeratosis of the epidermis and increased melanocyte accumulation within basal layer was seen

As the patient was noted to have a hypopigmented skin with silvery-grey hair, hair shaft examination and skin biopsy were done. Under the light microscopy, the patient’s hair showed irregular melanosome clumping along the shaft (Fig. 3A). The skin biopsy was generally normal (Fig. 3B), and EBER in situ hybridization was negative. Simultaneously, whole exome sequencing (WES) for genetic analysis was performed, which identified a novel homozygous mutation in *RAB27A* gene c.1 A > G (p.Met1Val, exon 2). Parents and his half-sister were found to be heterogynous for the same mutation.

Hence, the patient was diagnosed as GS2 with HLH, characterizing by CNS involvement at the onset. Therefore, etoposide-based HLH-1994 protocol for HLH were given. Recently, ruxolitinib (RUX), an oral selective JAK1/2 inhibitor, has shown great promise in mouse models of both primary and secondary HLH, including the improvements in central nervous system involvement [9–11]. RUX as a bridge to allogeneic hematopoietic stem cell transplantation (allo-HSCT) for refractory primary HLH is also reported [12]. Meanwhile, several clinical trials of RUX for HLH treatment are ongoing and the preliminary data suggest that RUX is active and safe in their settings. One of the enrolled patients was later diagnosed as primary HLH and achieved complete remission [13]. Therefore, we combined HLH-1994 regimen and RUX (5 mg, q12h) for this patient, which resulted in substantial resolution of all clinical symptoms and laboratory parameters. Within 3 days after HLH treatment, the patient became afebrile, and mental status recovered with fluid and neurological imaging abnormalities progressively improved. Repeat head CT showed his tonsillar hernia resolved by approximately 2 weeks of treatment, thus mannitol and glycerin fructose were weaned off. Furthermore, follow-up brain MRI at the 4th week showed most of the extensive hyperintense lesions were attenuated (Fig. 2). Hemoglobin and platelet count were progressively improved without further transfusion requirement by day 6. Inflammatory markers, including sCD25 and IFN-γ, had fallen rapidly to the normal levels. Repeat abdominal ultrasound showed complete recovery of hepatosplenomegaly. Then, he underwent haploidentical HSCT after 8 weeks of treatment with HLH activity in complete remission. At the time of writing, he has been discharged from the hospital and in good condition over three months.

## Discussion and conclusions

GS is a rare autosomal-recessive disorder with type 1, 2 and 3 distinct entities according to genetic involvement. The characteristic features of different types of GS and their involved genes are summarized in Table 2. Partial albinism affecting the hair and skin is a common feature to all the 3 types. Patients of GS1 have particularly neurological impairment and normal immune system, while GS2 is mainly characterized by immunological dysfunction with the development of HLH [3–5]. There have been also many reports showing CNS involvement in GS2, described variably as seizures, neuroinflammation, cerebellar ataxia, and increased intracranial pressure [7, 14–16].

**Table 2:** Characteristic Features of Griscelli Syndrome

	Griscelli Syndrome Type 1	Griscelli Syndrome Type 2	Griscelli Syndrome Type 3
Gene involved	*MYO5A*	*RAB27A*	*MLPH*
Inheritance	Autosomal recessive	Autosomal recessive	Autosomal recessive
Albinism features	Yes	Yes	Yes
Immune deficiency	Absent	Present	Absent
Hemophagocytic lymphohistiocytosis	Absent	Present	Absent
Neurological impairment	Severe (primary)	Rare (not primary)	Absent

In this case, hypopigmentation and severe neurologic manifestations were the marked clinical presentation resembling a GS1 phenotype. However, the symptoms and laboratory markers of HLH such as cytopenia, elevated sCD25 and low NK cell activity appeared as the disease progressed. It is likely that this patient presented with CNS HLH instead of the typical systemic HLH at onset. CNS involvement can be apparent at initial presentation, or it can occur at any time during the course of HLH, which include seizures, ataxia, facial palsies, spasticity, irritability, gait disorders, and coma [17, 18]. In patients without a positive family history, neurological deficits as the initial clinical markers may delay accurate diagnosis since the symptoms were similar to other neurological diseases, such as primary etiologies (e.g. autoimmune encephalitis and CNS vasculitis) and secondary etiologies (e.g. infections, tumor). This patient was initially suspected of unexplained infection, and underwent anti-infection treatment for long time without clinical improvement. The diagnosis was finally established with worsening clinical status and genetic testing. The presence of his hypopigmented skin and hair prompted the clinician to evaluate for HLH and gene screening. Recently, CNS involvement as an isolated manifestation of HLH has been reviewed in one study [19], which described the clinical spectrum of disease manifestations, response to therapy and prognosis in detail. In the study, pathological mutations were detected in *PRF1*, *RAB27A*, *UNC13D*, *LYST* and *STXBP2* with a mean interval to CNS HLH diagnosis of 28.3 months.

HLH-directed therapy followed by HSCT is the only curative treatment for patients with familial HLH, including GS2. According to a large retrospective study of HSCT in children with GS2, neurologic involvement before HSCT was a poor predictor of survival with a 5-year overall survival of 50 ± 12.5 % [20]. Our patient experienced a successful HSCT without neurologic sequelae, but the follow-up time of this patient is short, and the long-term survival and disease recurrence are still unknown. Several reasons may contribute to his successful HSCT: (i) the patient had no HLH activity and his CNS involvement had been greatly improved at the time of HSCT; (ii) Epstein-Barr virus (EBV) related lymphoproliferative disease is a main cause of failure HSCT for HLH, but he was free of EBV infection; (iii) RUX treatment before HSCT maybe helpful as reported in one study that RUX monotherapy served as an effective bridge to a second HSCT in one refractory HLH [12].

There are reports and studies that *RAB27A* mutations are associated with HLH but normal pigmentation, however these patients had different mutations from our patient [15, 21–23]. It is suspected that mutations at specific sites of *RAB27A* may selectively disrupt the interaction of RAB27A protein with UNC13D required for cytolytic granules secretion in lymphocytes, without impairing the interaction between melanophilin and Rab27a in melanocytes, explaining defective lymphocyte yet normal melanocyte function [21]. There are also studies elucidated novel structural variants at the noncoding region of *RAB27A* associated with an atypical form of GS2 characterized by marked HLH and normal pigmentation. It is hypothesized that lymphocytes and melanocytes might selectively use distinct *RAB27A* transcription start sites, with selective disrupting cellular cytototoxicity [22].

In conclusion, GS2 can have heterogenous clinical manifestations. This study emphasizes the need for greater awareness of subtle signs of partial albinism and CNS HLH in GS2, which are not infrequent in patients with *RAB27A* mutations. Early diagnosis and rapid control of the excessive inflammation with HLH-1994 regimen and ruxolitinib is the key to successful treatment for this patient. Besides, molecular diagnosis should be considered early in the diagnostic workup, which will be valuable for the prompt initiation of treatment and the reduction of late sequelae.

## Data Availability

The datasets used andanalyzed during the current study are available from the corresponding authoron reasonable request.

## Abbreviations

GS: Griscelli Syndrome
GS2: Griscelli Syndrometype 2
HLH: Hemophagocytic Lymphohistiocytosis
allo-HSCT: Allogeneic HematopoieticStem Cell Transplantation
CNS: Central Nervous System
RUX: Ruxolitinib
